# Right Bundle Branch Area Pacing in a Patient with Congenitally Corrected Transposition of the Great Arteries Using Three-dimensional Electroanatomic Mapping

**DOI:** 10.19102/icrm.2025.16012

**Published:** 2025-01-15

**Authors:** Serkan Cay, Hande Cetin, Serkan Topaloglu

**Affiliations:** 1 Division of Arrhythmia and Electrophysiology, Department of Cardiology, University of Health Sciences, Ankara City Hospital, Ankara, Turkey

**Keywords:** Corrected transposition, electroanatomic mapping, right bundle branch, pacing

## Abstract

A 35-year-old man with congenitally corrected transposition of the great arteries (ccTGA) and prior ventricular septal defect repair developed pacing-induced cardiomyopathy after 30 years of single-chamber VVI pacemaker use. Right bundle branch area pacing (RBBAP) was chosen for device upgrade due to its potential benefits over conventional cardiac resynchronization therapy. Advanced three-dimensional electroanatomic mapping was employed to navigate a synthetic patch from previous surgery and guide mid-septal electrode placement. Successful RBBAP was achieved with optimal parameters, complemented by atrial and defibrillator lead implantation. At 12 months, the patient showed improved ejection fraction (from 30% to 40%) and stable electrocardiographic results. This case illustrates the role of RBBAP and advanced mapping in addressing complex structural and conduction abnormalities in ccTGA.

## Case presentation

**Figure 1:**
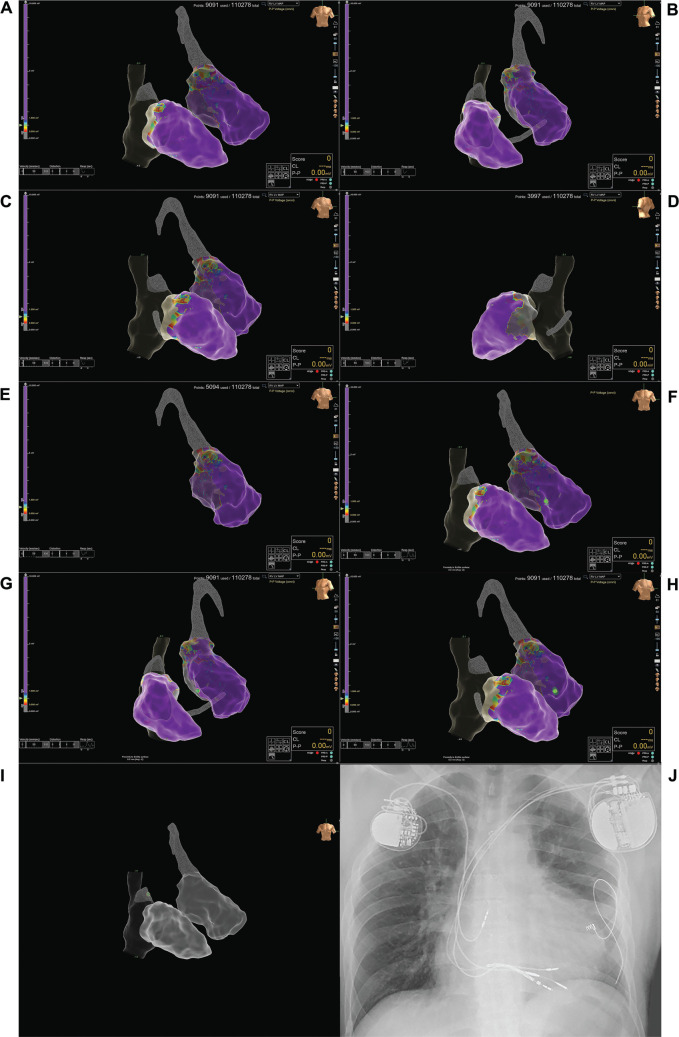
Three-dimensional voltage maps of both ventricles clearly depict the patch area in the basal septal region in the anteroposterior **(A)**, left anterior oblique **(B)**, and right anterior oblique **(C)** views. The patch area is also shown separately for the morphological left ventricle and morphological right ventricle in the posterolateral **(D)** and right anterior oblique **(E)** views, respectively. After implantation deep in the septum, the tip of the pacing electrode in unipolar configuration is seen as a green tag just beneath the scar area in the anteroposterior **(F)**, left anterior oblique **(G)**, and right anterior oblique **(H)** views. The tip of the atrial pacing electrode in unipolar configuration is observed as a green tag in the right atrial appendage **(I)**. A chest X-ray displays the left-sided implanted cardiac resynchronization device with the atrial, septal, and defibrillator electrodes. The right-sided older generator and pacing electrodes, endocardially on the right side and epicardially on the left side, are visible **(J)**. In the voltage maps, the gray area represents the right atrium, superior vena cava, and inferior vena cava, while meshed gray areas indicate the right atrial appendage and coronary sinus on the right side and the aorta on the left side.

A 35-year-old man, dependent on a pacemaker, with congenitally corrected transposition of the great arteries (ccTGA) (situs solitus) and a previously operated large ventricular septal defect, has had a VVI-mode single-chamber permanent pacemaker implanted for 30 years post-surgery. The initial implantation involved left-sided morphological right ventricular epicardial single-electrode unipolar pacing for 10 years. Subsequently, transvenous right-sided endocardial right-sided morphological left ventricular apical pacing was performed due to dysfunction of the epicardial electrode. He was referred to our division for a device upgrade due to pacing-induced cardiomyopathy of the left-sided morphological right ventricle. Given its inherent advantages over conventional cardiac resynchronization therapy, we opted for conduction system pacing using the right bundle branch area pacing (RBBAP) strategy. Due to a previous ventricular septal defect operation involving the use of a synthetic patch, three-dimensional electroanatomic mapping was performed before placing the pacing electrode to accurately locate the site of ventricular septal penetration. Using the EnSite™ X EP System (Abbott, Chicago, IL, USA) with omnipolar technology and the Advisor™ HD Grid Mapping Catheter (Abbott), we conducted three-dimensional mapping of the right and left heart structures. This mapping revealed a clear identification of the patch area, which manifested as a scar in the basal septal regions of both ventricles without any discernable retrograde His- or left-bundle potentials **(Figures 1A–1E, Videos 1–4)**.

**Video 1:** The Advisor™ HD Grid mapping catheter is observed during the mapping of the morphological left ventricle. Watch videos here: https://www.dropbox.com/scl/fo/vvqvgduw076pmuxjx22wh/ABJfHtZYTriz0JTd0TxD0jA?rlkey=cyhfzuaxlakk4js9esmj1ywrh&st=90s0tnkl&dl=0

**Video 2:** A 360° view of the right heart structures includes a voltage map and scar area of the morphological left ventricle. Watch videos here: https://www.dropbox.com/scl/fo/vvqvgduw076pmuxjx22wh/ABJfHtZYTriz0JTd0TxD0jA?rlkey=cyhfzuaxlakk4js9esmj1ywrh&st=90s0tnkl&dl=0

**Video 3:** A 360° view of the left heart structures includes a voltage map and scar area of the morphological right ventricle. Watch videos here: https://www.dropbox.com/scl/fo/vvqvgduw076pmuxjx22wh/ABJfHtZYTriz0JTd0TxD0jA?rlkey=cyhfzuaxlakk4js9esmj1ywrh&st=90s0tnkl&dl=0

**Video 4:** A 360° view of the right and left heart structures with voltage maps and scar areas of both the morphological left and right ventricles. Watch videos here: https://www.dropbox.com/scl/fo/vvqvgduw076pmuxjx22wh/ABJfHtZYTriz0JTd0TxD0jA?rlkey=cyhfzuaxlakk4js9esmj1ywrh&st=90s0tnkl&dl=0

**Figure 2:**
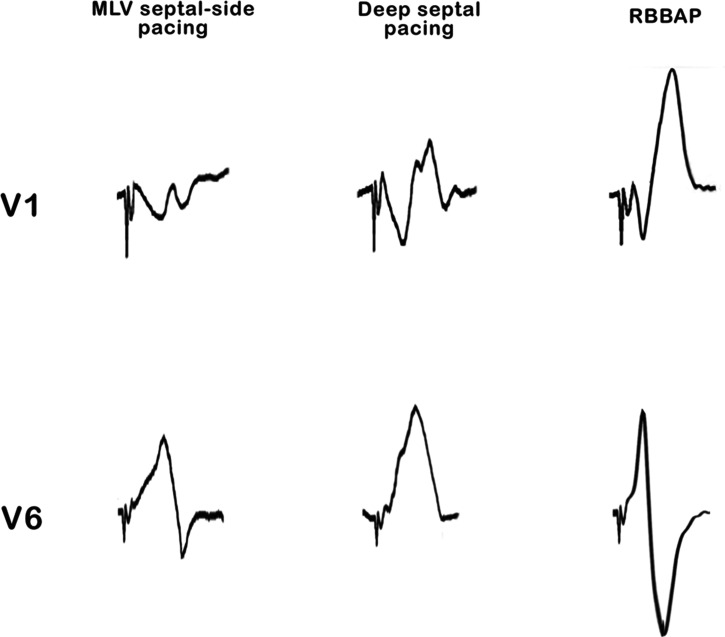
In the surface electrocardiogram during the progression of the septal pacing electrode in the interventricular septum, QRS changes can be observed in derivations V1 and V6.

Following this, an active-fixation pacing electrode (Solia S60; Biotronik, Berlin, Germany) was introduced from the left axillary vein through a pre-shaped delivery catheter (Selectra 3D; Biotronik) toward the morphological left ventricular side of the septum. Using a unipolar configuration, the pacing electrode was connected to both the pace/sense analyzer and the EnSite™ X EP System. By switching the electrophysiology mode to NavX™ (Abbott), the tip of the pacing electrode became visible in the preformed three-dimensional maps, appearing as a movable tag. With the assistance of both three-dimensional maps and fluoroscopy, we successfully achieved mid-ventricular septal placement of the pacing electrode just under the scar/patch area. Subsequent clockwise rotations of the pacing electrode from the shaft facilitated deep penetration toward the subendocardial region of the left side of the septum **(Figures 1F–1H, Videos 5 and 6)**. Unipolar pacing confirmed the capture of fibers of the right bundle branch, displaying a right bundle branch block morphology, negative concordance in the inferior leads, an R-wave peak time of 57 ms in lead V6, and an interpeak delay of 69 ms between leads V1 and V6. The QRS changes resulting in RBBAP were observed as the septal pacing electrode started from the entry site on the morphological left ventricular septal side, progressed first into the deep septal area, and then captured the conduction system on the morphological right ventricular septal side **(Figure 2)**. Pacing impedance and capture threshold were measured as 590 Ω and 0.9 V, respectively.

**Video 5:** Contrast septal angiography shows the deep septal penetration of both poles of the pacing electrode in the left anterior oblique projection. Watch videos here: https://www.dropbox.com/scl/fo/vvqvgduw076pmuxjx22wh/ABJfHtZYTriz0JTd0TxD0jA?rlkey=cyhfzuaxlakk4js9esmj1ywrh&st=90s0tnkl&dl=0

**Video 6:** A 360° view of the right and left heart structures includes voltage maps, scar areas of both the morphological left and right ventricles, and the tip of the pacing electrode located at the subendocardial region of the morphological right ventricle in the unipolar configuration, identified as a green tag. Watch videos here: https://www.dropbox.com/scl/fo/vvqvgduw076pmuxjx22wh/ABJfHtZYTriz0JTd0TxD0jA?rlkey=cyhfzuaxlakk4js9esmj1ywrh&st=90s0tnkl&dl=0

**Figure 3:**
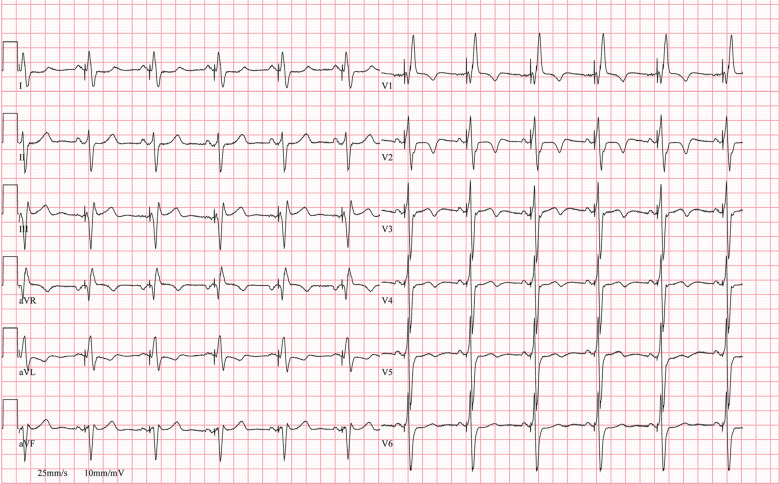
At the 12-month follow-up, the surface 12-lead electrocardiography reveals selectively stimulated right bundle branch, demonstrating right bundle branch block morphology with earlier activation of the lateral wall of the morphological right ventricle than the right septal endocardium of the morphological left ventricle.

**Figure 4:**
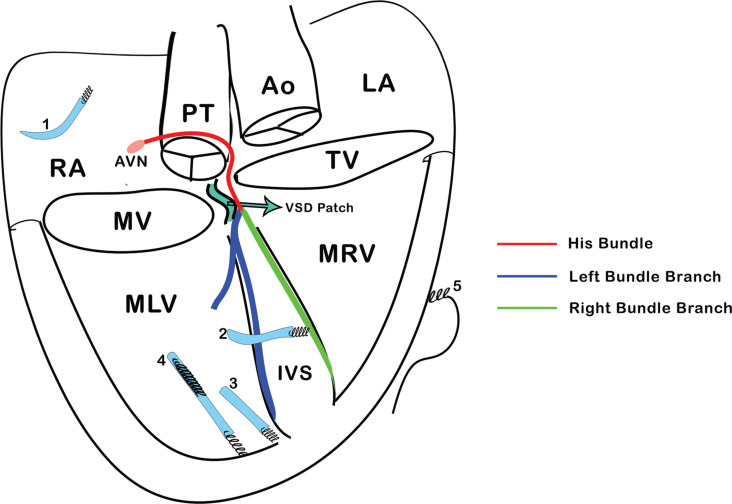
Gross anatomy of congenitally corrected transposition of the great arteries and ventricular septal defect. The His bundle, advancing from the front of the pulmonary trunk, then continues in the septal part of the morphological left ventricle (MLV) as the left bundle branch and in the septal part of the MRV as the right bundle branch. There are a total of five pacing electrodes: 1—within the right atrial appendage, 2—right bundle branch area pacing, 3—MLV apical, 4—MLV apical (implantable cardioverter-defibrillator), 5—MRV epicardial. *Abbreviations:* Ao, aorta; AVN, atrioventricular node; IVS, interventricular septum; LA, left atrium; MLV, morphological left ventricle; MRV, morphological right ventricle; MV, mitral valve; PT, pulmonary trunk; RA, right atrium; TV, tricuspid valve; VSD, ventricular septal defect.

Following this, an active-fixation electrode was implanted into the right atrial appendage, and it was also visible in the three-dimensional map in a unipolar configuration, appearing as a movable tag **(Figure 1I, Video 7)**. Finally, a DF-4 single-coil defibrillator electrode was implanted in the morphological left ventricle **(Video 8)**. A cardiac resynchronization therapy device was also implanted, with the atrial port connected to the atrial lead, the left ventricular port connected to the septal electrode, and the right ventricular sense/pace and defibrillator ports connected to the respective proximal tips of the defibrillator electrode **(Figure 1J, Video 9)**. Left ventricular–only pacing with the dual-chamber configuration was selected to enhance battery longevity. The patient declined extraction of the older pacing electrode, citing inherent risks, and thus, the electrode and the pulse generator were left in situ. At the 12-month follow-up, the ejection fraction had improved from the initial 30% before the upgrade procedure to 40%, and electrocardiography at this time showed the same findings as electrocardiography at implantation **(Figure 3)**. The gross anatomy of structural heart disease, the conduction system, and all implanted electrodes are seen in **Figure 4**.

**Video 7:** A 360° view of the right and left heart structures reveals the tip of the atrial pacing electrode located at the right atrial appendage in the unipolar configuration, marked with a green tag. Watch videos here: https://www.dropbox.com/scl/fo/vvqvgduw076pmuxjx22wh/ABJfHtZYTriz0JTd0TxD0jA?rlkey=cyhfzuaxlakk4js9esmj1ywrh&st=90s0tnkl&dl=0

**Video 8:** All implanted and older leads are visible, spanning from the right anterior oblique view to the left anterior oblique view. Watch videos here: https://www.dropbox.com/scl/fo/vvqvgduw076pmuxjx22wh/ABJfHtZYTriz0JTd0TxD0jA?rlkey=cyhfzuaxlakk4js9esmj1ywrh&st=90s0tnkl&dl=0

**Video 9:** The final positions of the generator and leads are shown in the antero-posterior projection. Watch videos here: https://www.dropbox.com/scl/fo/vvqvgduw076pmuxjx22wh/ABJfHtZYTriz0JTd0TxD0jA?rlkey=cyhfzuaxlakk4js9esmj1ywrh&st=90s0tnkl&dl=0

## Discussion

A significant proportion of patients with ccTGA develop dysfunction over time in the morphological right ventricle connected with systemic arterial circulation, typically manifesting during the fourth and fifth decades of life.^1^ Additionally, long-term pacing from the apex of the morphological left ventricle can adversely contribute to this process by causing dyssynchrony. In our relatively young patient, both conditions require consideration; although the ejection fraction has improved with conduction system pacing, complete normalization remains elusive. Optimal medical therapy for heart failure is also recognized to enhance ventricular dysfunction. Furthermore, according to our patient’s medical history, he has been receiving guideline-directed medical therapy, including newly introduced medications for heart failure, since the onset of symptoms of heart failure and determination of low ejection fraction.
